# Contribution of the ELRs to the development of advanced *in vitro* models

**DOI:** 10.3389/fbioe.2024.1363865

**Published:** 2024-04-08

**Authors:** María Puertas-Bartolomé, Desiré Venegas-Bustos, Sergio Acosta, José Carlos Rodríguez-Cabello

**Affiliations:** ^1^ Technical Proteins Nanobiotechnology, S.L. (TPNBT), Valladolid, Spain; ^2^ Bioforge Lab (Group for Advanced Materials and Nanobiotechnology), CIBER's Bioengineering, Biomaterials and Nanomedicine (CIBER-BBN), Edificio LUCIA, Universidad de Valladolid, Valladolid, Spain

**Keywords:** elastin-like, intrinsically disordered protein, *in vitro* model, bioprinting technology, organoid

## Abstract

Developing *in vitro* models that accurately mimic the microenvironment of biological structures or processes holds substantial promise for gaining insights into specific biological functions. In the field of tissue engineering and regenerative medicine, *in vitro* models able to capture the precise structural, topographical, and functional complexity of living tissues, prove to be valuable tools for comprehending disease mechanisms, assessing drug responses, and serving as alternatives or complements to animal testing. The choice of the right biomaterial and fabrication technique for the development of these *in vitro* models plays an important role in their functionality. In this sense, elastin-like recombinamers (ELRs) have emerged as an important tool for the fabrication of *in vitro* models overcoming the challenges encountered in natural and synthetic materials due to their intrinsic properties, such as phase transition behavior, tunable biological properties, viscoelasticity, and easy processability. In this review article, we will delve into the use of ELRs for molecular models of intrinsically disordered proteins (IDPs), as well as for the development of *in vitro* 3D models for regenerative medicine. The easy processability of the ELRs and their rational design has allowed their use for the development of spheroids and organoids, or bioinks for 3D bioprinting. Thus, incorporating ELRs into the toolkit of biomaterials used for the fabrication of *in vitro* models, represents a transformative step forward in improving the accuracy, efficiency, and functionality of these models, and opening up a wide range of possibilities in combination with advanced biofabrication techniques that remains to be explored.

## 1 Introduction

The search for *in vitro* models that faithfully reproduce the microenvironment of biological structures or processes holds great promise for understanding specific biological functions (Fatehullah et al., 2016). In the field of tissue engineering and regenerative medicine (TERM), replicating native extracellular matrix (ECM) is crucial to create *in vitro* models that accurately reflect the complexity of living tissues (Ibáñez-Fonseca et al., 2019; LeSavage et al., 2021). *In vitro* models have become invaluable tools for the study of cellular processes, disease mechanisms, and drug responses. The goal of *in vitro* models is to recreate bioengineered environments in a controlled and reproducible manner, with the highest possible fidelity, thus creating sophisticated platforms for basic research, but at the same time, powerful tools for high-throughput drug screening (Fu et al., 2020). This can also lead to a reduction in animal studies (Ingber, 2020). However, the efficacy of *in vitro* models depends on their ability to faithfully recapitulate the physiological conditions of the target tissues.

The correct selection of the biomaterial used to fabricate the *in vitro* model is fundamental. The ideal biomaterial must possess multiple features, such as biocompatibility, adaptability, mechanical robustness, and the ability to promote cell adhesion and proliferation. Additionally, it must adapt to the dynamic nature of living tissues, responding to signals and stimuli in the same way as the ECM, allowing cells to create their own matrix as close as possible to the native one. Achieving all these objectives is very difficult with both natural and synthetic polymers, encountering significant limitations (Frandsen and Ghandehari, 2012). Thus, biomaterials bioinspired on structural proteins emerge as a solution. Advances in synthetic biology and genetic engineering have paved the way for the evolution of biomimetic materials based on recombinant proteins with diverse applications in tissue engineering, biosensors, drug delivery, and theranostics (Acosta et al., 2020b; Varanko et al., 2020). In this context, elastin-like recombinamers (ELRs) are gaining importance due to their unique properties and versatile applications.

ELRs are protein-engineered polymers based on the repetition of low-complexity peptides found in tropoelastin (TE), the monomeric unit of native elastin. Elastin, a major component of the ECM, imparts elasticity and resilience to different organs and tissues such as skin, lungs and long blood vessels (Schmelzer and Duca, 2022). Being based on repeating TE motifs, ELRs mimic its mechanical properties and phase behavior. Importantly, ELRs are intrinsically disordered protein polymers (IDPPs) exhibiting a temperature-dependent liquid-liquid phase separation (LLPS), known as lower critical solution temperature (LCST) behavior (Roberts et al., 2015). This behavior allows to engineering modular molecular designs capable to hierarchically self-assemble into complex structures across various length scales (Quintanilla-Sierra et al., 2019; Saha et al., 2020). ELRs enable the control of order-disorder transitions of the synthetic matrix, facilitating the creation of dynamic systems with tailored functionalities and, overall, mimicking the intrinsic disorder found ECM components and intracellular intrinsically disordered proteins (IDPs) (Ulijn and Lampel, 2020).

**FIGURE 1:**
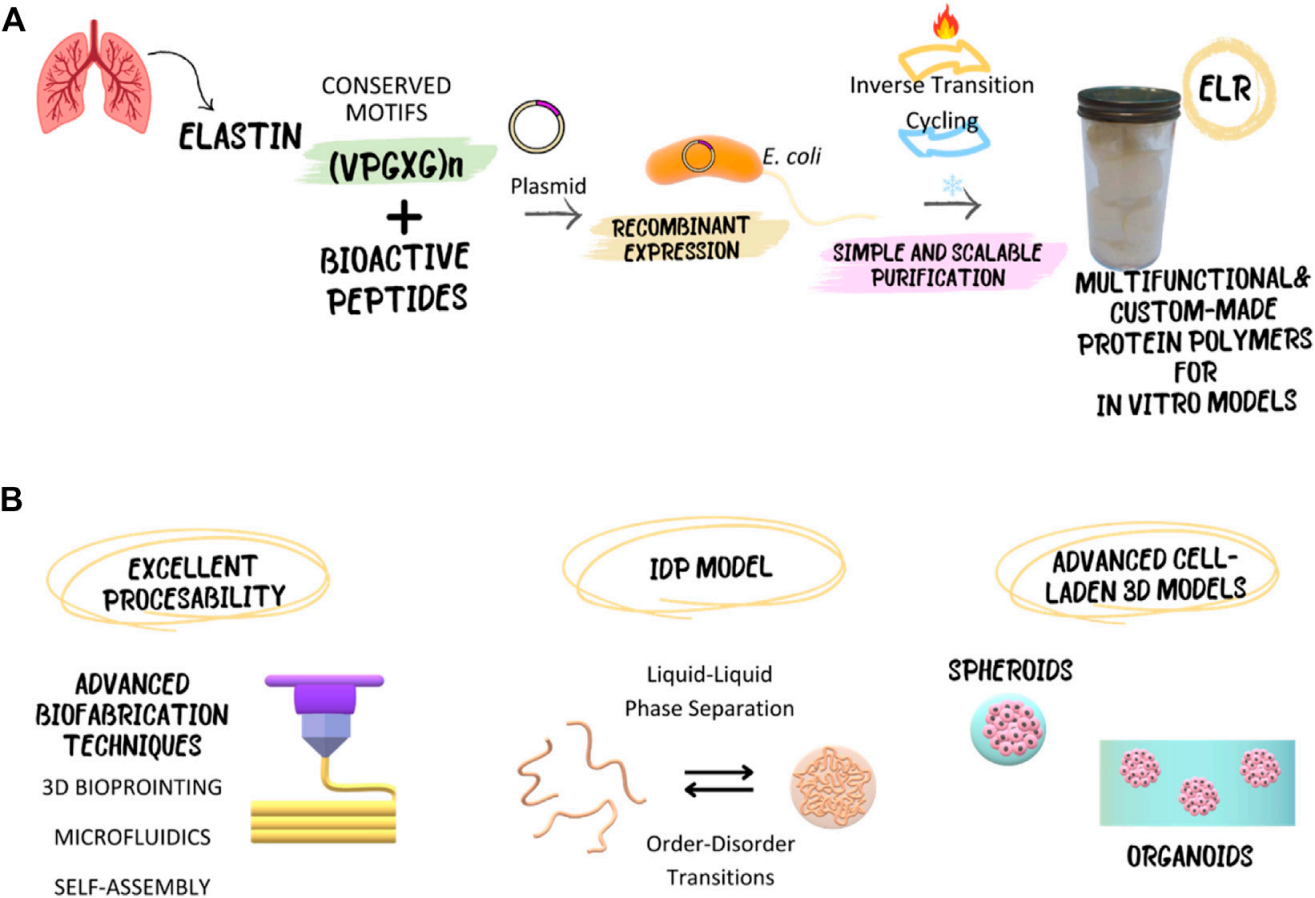
Elastin-like recombinamers (ELRs), with their recombinant and inherently disordered nature, enable the development of advanced *in vitro* models, both cell-laden and cell-free, serving as an intrinsically disordered protein (IDP) model. Additionally, their excellent processability allows for utilization through advanced biofabrication techniques. **(A)** Schematic representation of the recombinant production and purification of ELRs. **(B)** Overview of the possibilities that ELRs offer for the fabrication of innovative *in vitro* models.

In this sense, the sequence versatility and precise control in the synthesis of ELRs offer numerous advantages for the preparation of *in vitro* models, unmatched by synthetic and natural materials (Acosta et al., 2020b). Recombinant production ensures highly monodisperse and pure materials, coupled with a scalable and sustainable production system (Figure 1A). Moreover, their sequence is easily tunable and readily processable (Mbundi et al., 2021; Lima et al., 2022). Multivalent biocompatible polymers can be created with the ability to interact with multiple components (Sarisoy et al., 2023). They can be anchored to surfaces to enhance the biological response of inert materials or provide new functions (Costa et al., 2009; Li Y. et al., 2014; Acosta et al., 2020a; Alvisi et al., 2022). It is also possible to create complex cell instructive matrices adaptable to the needs of each tissue. By controlling the mechanical properties of the ELR matrix, as well as its biological properties, we can target the regeneration of tissues whose native ECMs have very different properties, including cartilage (Zhu et al., 2017; Cipriani et al., 2018), skin (Rodríguez-Cabello et al., 2018), cardiovascular (Fernández-Colino et al., 2019; Contessotto et al., 2021; González-Pérez et al., 2022; Saidy et al., 2022), or even neuronal and bone (Tejeda-Montes et al., 2014; Roth et al., 2023; Suhar et al., 2023). It is also possible to spatiotemporal control scaffold colonization as well as cell differentiation (Flora et al., 2019; González-Pérez et al., 2021; 2022). By introducing sequences sensitive to matrix metalloproteases with different kinetics it is possible to target complex regeneration processes such as neurogenesis and angiogenesis. Thus, multivalent *in vitro* models can be tailored fabricated with the ability to dynamically interact with multiple components and induce specific responses.

This review aims to comprehensively explore the contributions of ELRs to the development of advanced *in vitro* models. Our work goes beyond existing literature by providing a detailed discussion of the advantages of using ELRs, such as biocompatibility, tunability, and phase behavior, to elucidate the potential of application of these recombinant materials with unique properties to address the limitations of traditional models and contribute to the creation of more physiologically relevant models. In subsequent sections, we will elaborate on how their intrinsically disordered nature makes them interesting candidates as molecular models to study biological dynamic processes such as phase separation of biomolecular condensates, and how their ability to replicate decisive functions of the native ECM allows the creation of sophisticated *in vitro* models for regenerative medicine by different biofabrication techniques (Figure 1B). We anticipate this discussion will assist researchers in choosing ELRs-based biomaterials when developing new 3D *in vitro* models.

## 2 Bioproduction and purification ELRs

One of the primary advantages of ELRs, akin to other protein-engineered polymers, lies in their recombinant production (Wang et al., 2019). Leveraging recombinant DNA technology enables the meticulous customization of ELR sequences, facilitating the generation of virtually limitless ELR variants while ensuring exceptional purity, monodispersity, and exquisite sequence control (Acosta et al., 2020b). For the obtention of ELRs, distinct phases can be identified, encompassing gene construction, heterologous expression, and purification.

Regarding the challenge of producing polymeric genes, efficient methods have been developed. Initially, Urry and others employed type I endonucleases for gene insertion, generating lengthy constructs through random concatemerization (Guda et al., 1995). This method was based on “concatenation”, a random unidirectional ligation of ELR monomer genes through overlapping sticky ends, but lacked precise control over number and order of the gene fragments (Girotti et al., 2011). Subsequently, controlled monomer oligomerization methods emerged, such as iterative and recursive directional ligation (RDL). In RDL, short genes are combined in tandem, enabling the production of polymeric sequences in a precisely controlled and unidirectional manner, facilitated by the utilization of type IIS restriction endonucleases (Meyer and Chilkoti, 2002). Building upon RDL, Chilkoti and coworkers introduced recursive directional ligation by plasmid reconstruction (PRe-RDL). This process emerged to overcome problems like poor ligation efficiency, high background, and many time-consuming steps from RDL (McDaniel et al., 2010). Pre-RDL was utilized to rapidly clone ELR genes of any desired length and sequence (McDaniel et al., 2010), where two-halves of a vector, each containing the desired ELR gene, are ligated together, dimerizing the oligomer and reconstituting a functional plasmid. Furthermore, they developed a new random oligomerization method, combining rolling circle amplification with overlap extension polymerase chain reaction (OEPCR) (Ding et al., 2011; Girotti et al., 2011). This approach, known as overlap extension rolling circle amplification (OERCA), provides a one-step, rapid and highly parallel synthesis of genes encoding ELR polymers (Amiram et al., 2011). OEPCR, consists in a process that involves overlap elongation PCR amplification, but the nonspecific priming and mismatch pairing increased the error rate in gene sequences (Chu et al., 2011; Girotti et al., 2011). In the Chikolti laboratory, algorithms have been also developed to tackle the difficulty of synthesizing repetitive encoded sequences. They developed a codon-scrambling algorithm that exploits the codon redundancy of amino acids to find the least-repetitive synonymous gene sequence, enabling PCR-based gene synthesis of repetitive proteins (Tang and Chilkoti, 2016). Other laboratories have explored alternative methods. Xiong et al. explored the assembly and PCR-based accurate synthesis (PAS), which has been described as a simple and rapid technique. The authors of this method successfully synthesized DNA fragments up to 12 kb in length, containing high G + C content, repetitive sequences, and complex secondary structures (Xiong et al., 2006). Additionally, a more efficient technique has been developed recently for producing long chains of ELRs. This method involves successive rounds of Gibson assembly, which almost doubles the ELR length with each cloning iteration (Deyling et al., 2018). Thus, several methodologies are available for the synthesis of ELR genes.

Regarding ELR bioproduction, *E. coli* is the most widely used organism for heterologous expression. *E. coli* has been widely utilized for recombinant protein production due to its rapid growth rate, simple and optimized growth conditions, ease of genetic manipulation, high product yield, and scalability (Burnett and Burnett, 2020; Lima et al., 2022). However, eukaryotic organisms such as plants have been explored for ELR expression due to the advantages they present (Lima et al., 2022). Yeast provides advantages such as simple genetic manipulation, simple and cheap growth conditions, well-characterized cell lines, and can be easily adapted to industrial-scale conditions. In contrast to bacteria, yeasts present the ability to perform adequate post-translational modifications (PTMs) such as glycosylation or methylation, after translation. Arginine methylation is important for regulating phase separation and can alter the charge distribution and hydrophobicity (Owen and Shewmaker, 2019), while nonhuman glycosylation can cause an immune response against the recombinant proteins and can alter function, solubility, and stability (Lim et al., 2010). For this reason, PTMs are an important concern for the different expression systems. Plants can produce complex proteins. They present the maximum scalability, low cost, absence of endotoxins, and lack of human pathogens (Twyman et al., 2003; Basaran and Rodriguez-Cerezo, 2008; Burnett and Burnett, 2020). However, plant expression systems lack regulatory approval and present nonhuman PMTs (Burnett and Burnett, 2020).

For ELR purification, it is important to note their reversible LCST phase behavior (Li N. K. et al., 2014) Below its LCST, ELR chains are well-solvated and, as a consequence, highly soluble in aqueous solution. When the solution is heated and the LCST is reached, ELRs become insoluble, form large micron-size aggregates, and precipitate out of the solution, resulting in high yields of the target protein with high purity (Luan et al., 1990; Meyer and Chilkoti, 2002). This transition is completely reversible so that the aggregated polypeptide completely dissolves when the temperature is lowered below the LCST of the ELR. This phase behavior enables easy purification through heating and cooling cycles (Meyer and Chilkoti, 1999). This method (Figure 1A), termed *Inverse Transition Cycling* (ITC), is a cost- and time-efficient eliminating chromatography and therefore, limitations by resin capacity. Additionally, ELRs can be used as purification tags for target proteins or peptides, further simplifying the purification process (Hassouneh et al., 2010). For instance, ELR has been fused to human epidermal growth factor (hEGF) (Sarvestani et al., 2021), antimicrobial peptides (Acosta et al., 2020c; Pereira et al., 2021), bioactive human interferon-gamma (IFN- γ) (Heidari-Japelaghi et al., 2020), and As(III) S-adenosyl-methyltransferase (ArsM) (Ke et al., 2019), among others, illustrating the potential to incorporate growth factors, antimicrobial peptides, interleukins, and enzymes into our *in vitro* models.

## 3 ELRs as molecular models of intrinsically disordered proteins

The evolving field of LLPS in proteins, as the basis of biomolecular condensates formation, has motivated the study of IDPs and their remarkable dynamic properties. Biomolecular condensates are membraneless intracellular structures that play a crucial role in the organization of cellular behaviors. These condensates, arising from the spontaneous phase separation of IDPs, create systems that compartmentalize multiple cellular functions simultaneously (Chen and Silver, 2012). The intrinsic structural disorder not only enables dynamic interactions with other cellular components but also imparts unique mechanical properties, as seen in structural proteins like TE and resilin (Dzuricky et al., 2018). Thus, ELRs have served as *in vitro* models, shedding light on the mechanics and molecular variables regulating complex biological processes associated with protein phase separation and protein disorder-order transitions (Ligorio and Mata, 2023), such as elastogenesis, biomolecular condensate formation, or biomineralization processes.

### 3.1 ELRs as *in vitro* models of biomolecular condensates

The ability of protein polymers derived from elastin to undergo LLPS (Dzuricky et al., 2018), presents a unique potential to create *in vitro* systems for studying the principles that govern the formation of biomolecular condensates, but also opens the possibility to create innovative synthetic condensates with customized topology, mechanical properties, or chemical nature (Dai et al., 2023b).

The recombinant polymeric nature of ELRs places them at the converging point of synthetic biology and polymer science. Harnessing synthetic biology tools allows the creation of recombinant polymers with precisely tailored phase transitions, facilitating the incorporation of unnatural amino acids and post-translational modifications (Costa et al., 2018; Mozhdehi et al., 2018; Scheibel et al., 2020). Concurrently, the application of polymer science principles enables the rational design of modular self-assembling systems and their bioconjugation with other polymers (Acosta et al., 2020b; Saha et al., 2020; Garanger and Lecommandoux, 2022).

ELRs present an opportunity to uncover the variables controlling the intricate LLPS processes occurring in more complex IDPs. For instance, the generation of microdroplets using polymers via microfluidics allows the creation of cell-free systems that mimic intracellular molecular crowding and offers a means to study phase transitions (Liu et al., 2020). In these *in vitro* models, it has been observed that diverse molecular assembly patterns can be achieved (Simon et al., 2017). The combination of ELRs with different hydrophobicities, i.e., transition temperatures, allows for the creation of multicompartimentalized microenvironments. It has been observed that the mixing properties of ELRs conform to the Flory-Huggins mean-field theory, enabling the understanding and prediction of the miscibility or immiscibility of ELR-based coacervates based on molecular parameters such as chain length, composition, and interaction with the solvent and between ELRs (Simon et al., 2017).

Microfluidics has enabled the creation of paired emulsions from immiscible polymers, such as dextran and poly (ethylene glycol) (PEG) to simulate crowded molecular environments (Zhao et al., 2020). These synthetic systems have served to investigate phase separation and spatial distribution of ELRs in confined environments with multiple phases, where ELR coacervation preferably occurs at the interface of the two phases (Zhao et al., 2020). This type of synthetic cell-like system allows also to study the dynamic coacervation of ELRs and the combination with other proteins, and can serve as a platform to evaluate interactions of IDPs with other cellular components. Indeed, in a different study, monoblock ELRs were covalently bioconjugated with dextran and PEG to study the mechanisms of co-assembly. Once again, it was demonstrated that ELR coacervates preferably form at the interface of both phases, and that this co-assembly mechanism was reversible (Zhao et al., 2021). Moreover, it has been demonstrated that synthetic condensates based on bioconjugated ELRs exhibit a remarkable capability to enhance the rate of enzymatic reactions. By creating cytomimetic protocellular models using water-in-oil-in-water (W/O/W) double emulsions and employing monoblock ELRs and ELR-bioconjugates with PEG and horse radish peroxidase as a model multicomponent enzymatic system, a study model for the kinetics of enzymatic reactions under different osmotic pressures was established. This approach demonstrated that synthetic condensates significantly improved the rate of enzymatic reactions after hypertonic shock (Schvartzman et al., 2023).

The versatile design of ELRs enables the production of protein polymers with multifaceted functionalities. It is feasible to create *in vitro* models of biomolecular condensates induced by variables other than temperature, such as pH. ELRs containing charged amino acids in their sequence are responsive to pH changes (Ribeiro et al., 2009; MacKay et al., 2010). By controlling the proportion of charged (i.e., Glu and His) and hydrophobic (i.e., Val, Ile, Phe and Tyr) amino acids in the guest position, de Haas et al. engineered pH-responsive ELRs with the capability to form condensates under narrow pH changes within a physiologically relevant range (i.e., pH 4–7) (de Haas et al., 2023).

Moreover, ELRs can be modified at the sequence level to incorporate other protein domains capable of interacting with other cellular components, thus developing models of multivalent condensates. Since one of the roles of biomolecular condensates is the control of gene expression, the interaction of ELR-based condensates with nucleic acids by introducing nucleic acid-binding domains allows the construction of intricate systems capable of elucidating mechanistic variables in the aggregation of more complex natural ribonucleoproteins (Simon et al., 2019). Thus, opening avenues for designing synthetic condensates applicable to metabolic engineering (Qian et al., 2022).

However, it is noteworthy to consider that synthetic models have limitations, since the study conditions may differ from intracellular conditions. Nevertheless, it is possible to transfect living cells with genes encoding protein polymers and express them directly intracellularly to create even more precise *in vitro* models. These approaches facilitate the examination of their interactions with intracellular components in both eukaryotic and prokaryotic living cells, thereby establishing more intricate condensates (Dai et al., 2023a). These sophisticated models can serve as potent tools in synthetic biology for cellular engineering applications. In this way, the capacity of amphiphilic block co-ELRs to program self-assembly in living *E. coli* cells was investigated (Huber et al., 2023). From a large library of building blocks, crucial molecular variables were unveiled for programming self-assembly, including charge, distribution, and hydrophobic strength. This demonstrated that depending on the composition of ELRs, the derived condensates are capable of influencing the distribution and spatial organization of DNA, potentially guiding molecules into synthetic condensates.

The creation of synthetic condensates from ELRs has also served to shed light on other biological processes where LLPS is involved, such as elastogenesis. Elastogenesis, the formation of elastic fibers involves a harmonious orchestration of TE synthesis, coacervation, crosslinking, and deposition on microfibril scaffolds (Yeo et al., 2011). The first step of elastogenesis is the condensation or coacervation of TE (Tu et al., 2010). Understanding this process is crucial for comprehending pathologies related to poor elastic fiber deposition and for engineering functional materials that accurately mimic elastic matrices (Tarakanova et al., 2018).

ELRs inspired by representative TE domains emerged as invaluable tools for *in vitro* modeling, particularly in the early stages of elastogenesis. Multiple elastin-like polypeptides have been produced, using different TE exons as repeat units, with those located in the hinge region (i.e., exons 20, 21, 23, and 24) being among the most recurrently employed (Muiznieks and Keeley, 2010; Reichheld et al., 2014). These exons, combining hydrophobic and hydrophilic elements, play a crucial role in the structural flexibility of TE (Yeo et al., 2016), presenting a simplified but representative version of the full protein (Reichheld et al., 2017). Through the permutation of these exons, Muiznieks et al. developed ELRs with different coacervation capacities. These models allowed the *in vitro* study of specific mutations in different ELR domains and evaluated their effect on coacervate formation. The meticulous control of coacervate (condensate) droplet growth and stability stands as a prerequisite for the proper maturation of elastic fibers. Understanding the formation of these coacervates holds significance from both physiological and biomaterial science perspectives. In this way, these models allowed the investigation of coacervation events, aggregation at interfaces, and co-localization with other proteins involved in elastogenesis, such as Fibrillin-1 (Muiznieks et al., 2014). While molecular weight and hydrophobicity of the recombinant polypeptides are crucial modulators of the initial phase separation, other factors influence the maturation of the formed liquid coacervate. Hydrophobic elastin domains initiate phase separation, but the addition of charged lysine residues in crosslinking domains introduces an amphipathic character, influencing droplet stability. These protein polymer approaches can also facilitate the emulation of crosslinking effects *in vitro*, providing insights into the intricate interplay between coacervation and crosslinking processes (Lau et al., 2022).

Condensate properties can be controlled by sequence changes in the ELRs. While TE can exhibit LLPS, Ceballos et al. have demonstrated that the production of recombinant polymers bioinspired in TE exons 20–21-23–24, together with an increase of the hydrophobic content in their terminal domains, has the potential to alter the condensate maturation. This alteration leads to liquid-solid transitions in saline solutions providing a valuable avenue for modulating elastogenesis processes *in vitro* (Vidal Ceballos et al., 2022).

Moreover, the creation of models from ELRs enables the monitoring of coacervation processes with enhanced resolution (Reichheld et al., 2017). Thanks to the use of ELRs, it has been experimentally demonstrated, employing solution and solid-state NMR, that the hydrophobic domains of TE are intrinsically disordered, demonstrating their enormous potential to mimic complex *in vitro* phase transition processes and study them in detail in a controlled manner that allows replication.

### 3.2 ELRs as *in vitro* models for biomineralization

Biomineralization is the natural process through which living organisms create minerals by depositing inorganic molecules using organic molecules as scaffolds, thus creating biocomposite materials such as bone and enamel (Aparicio and Ginebra, 2015). Depending on the type of organism, different biohybrid structures can be generated by depositing various minerals, including magnetite, silicates, or hydroxyapatite (Boskey and Villarreal-Ramirez, 2016). Particularly relevant from a pathological and biomaterial science perspective is the deposition of calcium phosphate on macromolecular biological structures. In this context, IDPs play a crucial role, as their structural flexibility allows them to interact with ions in solution, leading to conformational changes that enable them to modulate and inhibit *in vivo* biomineralization (Boskey and Villarreal-Ramirez, 2016). Even in the case of bones, where collagen type I, a protein with a defined secondary structure, serves as a scaffold for the formation of mineralized tissue with apatite crystals (Beniash, 2011), IDPs control the deposition and maintenance of biomineral (Boskey and Villarreal-Ramirez, 2016). Thus, protein components of the extracellular matrix with high proportions of disordered sequences will play an important role in ECM aging and calcification processes and may in turn prove to be a revolutionary source of inspiration for the creation of innovative biomineralized materials.

Arterial calcification, characterized by the deposition of calcium phosphate (Ca–P) minerals on the ECM of arteries, is a pathological condition associated with increased morbidity in patients with chronic kidney disease, type 2 diabetes, atherosclerosis, and certain genetic disorders (Shanahan et al., 2011; Heinz, 2021; Schmelzer and Duca, 2022). Several studies have analyzed minerals formed in cardiovascular tissues (Danilchenko et al., 2013; Cottignoli et al., 2015), and found that the calcification process may resemble the multistep mechanism observed during the formation of the mineral component of bone (Gourgas et al., 2018). Arterial calcification is marked by the initial adsorption of Ca^2+^ ions, the progressive formation of calcium phosphates, and their subsequent transformation into hydroxyapatite and carbonated hydroxyapatite (Gourgas et al., 2018). However, further studies are necessary to validate these findings.

**FIGURE 2:**
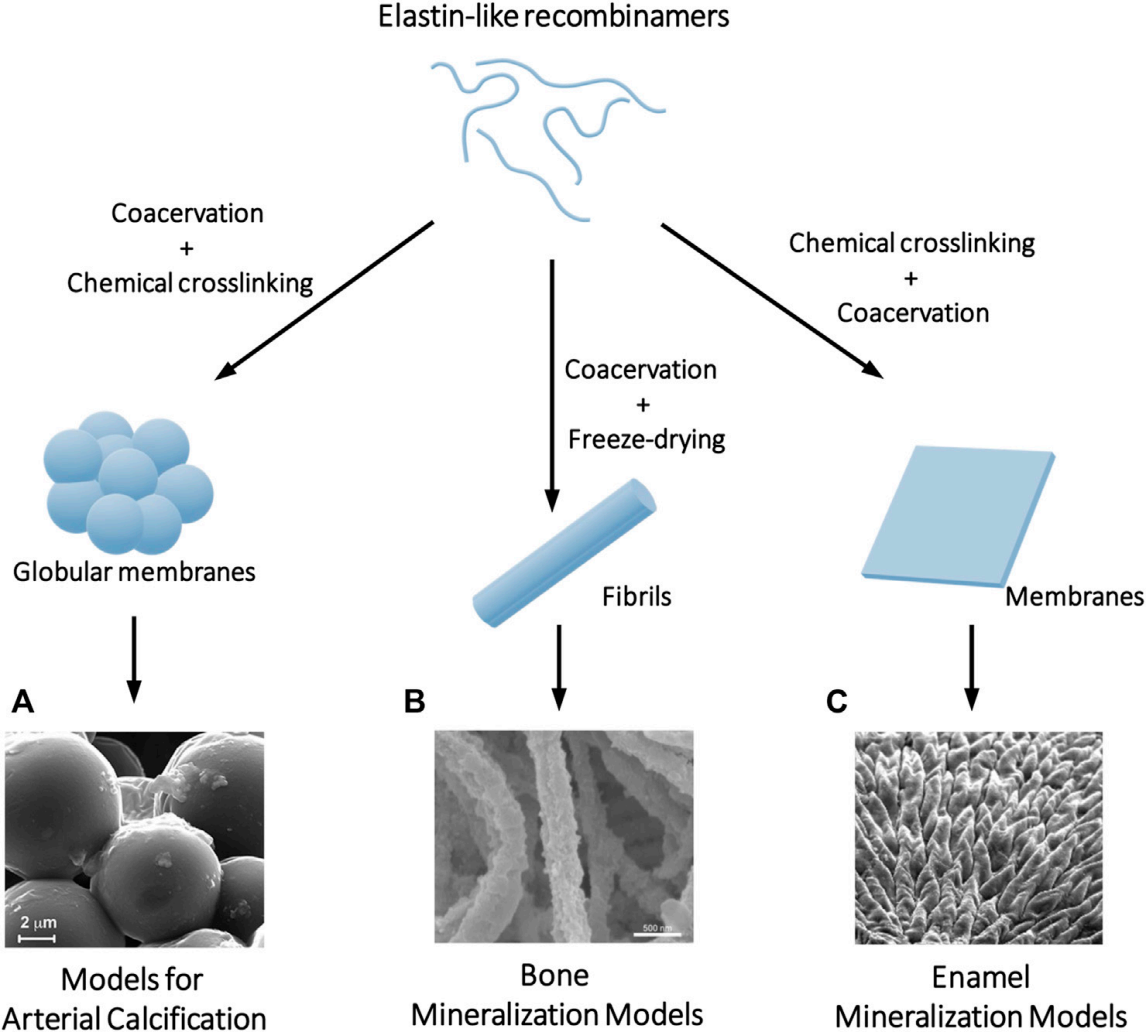
Examples of biomineralization *in vitro* models where ELRs serve as a scaffold to control and direct crystal nucleation and growth. Depending on the processing of the ELRs, different topographies can be achieved including **(A)** globular membranes that serve as a calcification model of the ECM of the arteries, **(B)** fibrils that simulate the mineralization process that occurs in collagen fibers in bone tissue, or **(C)** membranes where chemical crosslinking controls the order-disorder ratio and thus the hierarchical mineralization of the material can be programmed in the same way as it occurs in the enamel. Adapted with permission from references (Li et al., 2017; Elsharkawy et al., 2018; Gourgas et al., 2019b).

*In vitro* models offer a valuable approach to study arterial calcification. Early models, utilizing solubilized elastin fragments, allowed to elucidate the biochemical principles of calcification (Starcher et al., n. d.; Sobel et al., 1966). However, these models had significant limitations, including poor solubility, handling difficulties, processability issues, and the presence of impurities and contaminants that could impact results. In contrast, ELRs have emerged as precise *in vitro* elastin models for calcification. ELRs address the limitations of earlier models, allowing the creation of systems from highly pure, composition-controlled, and tailor-made materials, allowing high reproducibility. Particularly notable contributions have been made by Cerruti and coworkers, who, using recombinant polymers based on cross-linking and hydrophobic domains TE exon repeats, allow for controlled self-assembly and cross-linking to form biomimetic gels and membranes (Figure 2). These membranes allowed the development of an *in vitro* model for medial calcification (Gourgas et al., 2019b; 2019a; Parashar et al., 2021; Lau et al., 2022).

The model, employing cross-linked ELR membranes immersed in simulated body fluid (SBF), replicates mineral phase evolution observed in mouse models. This allows an understanding of the topographical factors influencing crystal formation (Gourgas et al., 2019b), the creation of models of calcification adapted to different pathological conditions (Gourgas et al., 2019a), and the study of interactions with calcification inhibitors (Parashar et al., 2021). Although it proves to be a promising model as a potential drug screening platform, future directions involve optimizing coacervation and cross-linking conditions to enhance biomimicry, enabling a more comprehensive understanding of medial calcification processes and the development of potential treatments.

While the ability of elastin to calcify poses a significant medical challenge, it has also been employed to create biohybrid innovative materials with properties mimicking those of natural mineralized tissues that may serve as bone or enamel *in vitro* models. One critical property for controlling mineral deposition is the degree of structural disorder and order in the proteins of our scaffold and their capacity to form hierarchical assemblies (Elsharkawy and Mata, 2018). The interaction of inorganic ions with the disordered regions of the protein leads to its transition to more ordered structures, and this conformational reorganization will mark the nucleation and growth of the crystal.

In the initial studies aimed at *in vitro* production of organic-inorganic composites from ELRs, a biomimetic approach was chosen based on incorporating the peptide DDDEEKFLRRIGRFG into the ELR backbone at the sequence level. This peptide corresponds to the SNA15 domain of statherin, a salivary IDP that regulates calcium and phosphate mineralization of enamel (Gil Mur, 2016). However, despite the ability of statherin-derived peptides to promote mineralization in ELR membranes, hydrogels, or coatings (Li Y. et al., 2014; 2015; Tejeda-Montes et al., 2014; Shuturminska et al., 2017), it was observed that the self-assembly capacity of ELRs played a crucial role in promoting crystal nucleation. Studies with ELRs in solution already showed that molecular designs leading to supramolecular aggregates in suspension had a greater capacity to induce crystal nucleation (Prieto et al., 2011; Misbah et al., 2016). The crystal morphology could be guided depending on their ability to self-assemble into defined nanostructures (i.e., micelles) or amorphous coacervates. Later, Aparicio and coworkers studied the mineralization of ELR fibrils *in vitro* using the polymer-induced liquid-precursor (PILP) process (Li et al., 2017). They compared the ability to achieve intrafibrillar mineralization in ELRs with different sequences and observed two highly relevant events. Firstly, the coacervation of ELRs was necessary to induce mineralization, and secondly, fibers derived from ELRs without bioactive sequences, once mineralized, were much more stable than those presenting different functional motifs, whether cell-adhesive or statherin-derived peptides. This demonstrated that the introduction of charged peptides into the ELR backbone determines its self-assembly and will alter its balance between ordered and disordered structures, directly impacting the *in vitro* mineralization of the protein material.

Mata and coworkers systematically studied the control of the degree of structural disorder and order in self-assembled ELR membranes through chemical crosslinking (Elsharkawy et al., 2018). Modulating the degree of disordered structures allows controlling and programming the calcium phosphate nucleation and hierarchical growth of hydroxyapatite crystals, similar to what occurs in dental enamel. This way, mimicking its mechanical properties is achieved. Moreover, this process can be applied to different surface topographies, creating customized models of mineralized tissue. The tunability of the elastin-like matrices together with the nanotopography enables precise spatiotemporal control of the directionally guided growth of hierarchically organized mineralized structures over millimeter length scales, which has implications for developing biomimetic models with advanced functionalities for applications in materials science and tissue regeneration (Deng et al., 2021).

## 4 ELRs for 3D *in vitro* models in regenerative medicine

The conventional *in vitro* study model employing a monolayer of cells in a Petri dish, has significantly contributed to biological research, but falls short in representing *in vivo* cellular interactions (cell-cell and cell-ECM interactions) (Ferreira et al., 2018) (Costa et al., 2016), which play a pivotal role in altering cell behavior, impacting differentiation, proliferation, signaling, and responsiveness to stimuli (Agrawal et al., 2020). This conventional model exposes all cells uniformly to nutrients and growth factors, overlooking the dynamic distribution seen in diseases like cancer, hindering realistic *in vivo* cell condition mimicry (Mao et al., n. d.). To overcome these constraints, biofabrication and regenerative medicine are actively advancing materials and structures that replicate the native ECM, enabling the creation of highly precise 3D tissue models for systematic investigations. 3D models closely resemble native conditions, offering a diverse set of biochemical and biophysical cues that maintain the differentiation capacity of stem cells, enabling self-renewal and self-organization into complex structures (Hofer and Lutolf, 2021).

### 4.1 ELRs for *in vitro* spheroids models

Spheroids have been widely used in TERM as *in vitro* 3D models. These 3D structures present high cell-cell and cell-ECM interactions enhancing cell viability, protein secretion, and stem cell differentiation due to the increased chemical/mechanical interactions (Kim et al., 2020). The culture methods employed for spheroid fabrication maximize cell-cell interactions (Caprio and Burdick, 2023) and can be broadly divided in scaffold-free and scaffold-based approaches. Scaffold-free approaches, such as hanging drop, magnetic levitation, pellet culture, or spinner flask techniques, have been broadly used to construct spheroids (Decarli et al., 2021). However, these methods involve labor-intensive processes, low throughput, and difficulty in spheroid size control. Consequently, scaffold-based approaches using functional biomaterials have been developed to produce high-throughput, homogeneous spheroids (Ryu et al., 2019). Scaffold-based approaches can be roughly divided into hydrogels, where cells are embedded into the scaffold, and solid scaffold, where cells are seeded atop the scaffold (Table 1) (Knight and Przyborski, 2015; Lee and Lee, 2022).

**TABLE 1:** Techniques for spheroid fabrication using ELRs, either as solid scaffolds with cells seeded onto them, or as hydrogels with cells embedded within. The table also details the specific ELRs biomaterial types employed for spheroid fabrication and the observed results.

Scaffold- based approaches	Biomaterial		ELR composition	Observations		References
Solid-scaffold	ELR-PEI^a^ (positively charged)	ELR-PEI800 (low aminated surface)	(VPGVG)_40_ ELR chemically conjugated to PEI (M_w_ = 800 Da)	Cells formed cellular aggregates within 48 h due to the interaction of the polymer coating with the cell membrane	Formation of spheroids with an average diameter ∼100 µm hASCs exhibited superior osteogenic and adipogenic differentiation, when supplemented with differentiation media, compared to 2D monolayer	Adams et al. (2014), Gurumurthy et al. (2017), Turner et al. (2017), Fitzgerald et al. (2020)
		ELR-PEI25000 (high aminated surface)	(VPGVG)_40_ ELR chemically conjugated to PEI (M_w_ = 25,000 Da)		Formation of spheroids with an average diameter ∼50 µm	Fitzgerald et al. (2020)
	ELR-PAA^b^ (negatively charged)		ELR with a (VPGVG)_40_ composition was chemically conjugated to PAA using carbodiimide chemistry	Adhesive interaction between cell-surface. Cells seeded spread and formed a monolayer without spheroid formation Negatively charge surfaces contribute to cell-substrate interaction, in contrast to positively charge surfaces where cell-cell adhesive forces seems to be greater than between cells and substrate		Janorkar et al. (2008)
	ELR-RGD		Alternating elastic (VGVPG)_6_ structural domains and the fibronectin-derived Arg-Gly-Asp (RGD) sequence	Clustering of β-cells into pseudoislets and improvement in insulin release compared to that of monolayer culture		Jeon et al. (2011), Jeon et al. (2012), Lee et al. (2013)
Hydrogels	ELR-collagen		Different ratios of ELR (with a primary sequence of (VPGVG)_120_) to collagen. Scaffolds formed by mixing both polymers	ELR-collagen scaffolds showed superior mechanical attributes (elastic moduli of 11–18 MPa) than pure collagen scaffolds (elastic moduli of 2–4 MPa)		Amruthwar et al. (2013)
			Scaffolds created either blending ELR and collagen solutions or by crosslinking them with EDC	Stiffer and/or crosslinked elastin-collagen based scaffolds constricted the spreading of hASCs, resulting in a spheroid morphology and promoting an enhanced osteogenic or adipogenic differentiation		Pal et al. (2019), Newman et al. (2020a)
	ELR-decorated with bioactive peptide		Single-step, cell-compatible method to tether QK^c^ peptide into ELR hydrogels	Soluble QK, when no tethered, diffuses away from the site of action and escapes from the hydrogel. In contrast, by tethering the QK peptide to the ELR hydrogel, concentrations of QK were sustained, resulting in prolonged angiogenic signaling and enhancing endothelial outgrowth		Cai et al. (2014)
	HE5-C + HRGD6-N_3_ hydrogel		Hydrogels were fabricated using two distinct ELRs: one featuring the RGD cell adhesion motif (HRGD6), and the other incorporating metalloproteinase-cleavage domains (HE5)	When enclosed within ELR hydrogels, both the non-metastatic cell line and non-tumorogenic breast epithelial cells exhibited spheroid formation		Blanco-Fernandez et al. (2022)

^a^ PEI: polyehyleneimine.

^b^ PAA: polyacrilic acid.

^c^ QK: angiogenic peptide.

The encapsulation of cells in a hydrogel, a popular option for 3D cultures (Tibbitt and Anseth, 2009), can be designed to support specific types of cell growth and function by encapsulating cells in an artificial ECM environment that promotes spheroid formation (Heywood et al., 2004; Topman et al., 2013). Cells can be encapsulated into the gels through various methods, including self-assembly, radical polymerizations induced by UV exposure, as well as ionic or chemical cross-linking. In contrast, seeding cells onto coated surfaces entails the prior application of biomaterial solid scaffolds, followed by cell deposition atop the scaffold (Knight and Przyborski, 2015). Solid scaffolds may feature specific patterns conducive to spheroid formation in designated areas, promoting tissue-like mass aggregation and enhancing cell-cell interactions over cell-surface interactions (Ursini-Siegel, 2023).

These scaffold-based approaches may consist of natural materials such as fibrin, hyaluronic acid, collagen, or Matrigel, as well as synthetic materials like PEG and poly (-caprolactone) (PCL) (Lee and Lee, 2022). Drawbacks observed in biological matrices like Matrigel (i.e., poor mechanical properties, inherent variability, and high sensitivity to enzymes) or in synthetic polymers (i.e., biofunctionalization requirement, poor biocompatibility, slow water absorption, and poor toughness) underscore the necessity for the development of a biocompatible, reproducible, and cost-effective matrix with control over mechanical and chemical properties (Wu X. et al., 2021; Wu Y. et al., 2021).

The use of ELRs offers multiple advantages over the traditional materials used for designing an optimal matrix for spheroid fabrication in a scaffold-based approach. ELRs allow the construction of an adaptable platform with a high degree of fabrication control over scaffold properties such as porosity, surface polarity, mechanical strength, and rate degradation (Fernández-Colino et al., 2018; Fernández-Colino et al., 2018; Wang et al., 2018; Wang et al., 2018; Flora et al., 2019; Flora et al., 2019; Shayan et al., 2023; Shayan et al., 2023). For instance, although limited diffusion in hydrogels has proven advantageous in fields such as controlled release of drugs and nutrient delivery in agriculture, it can hinder the prolonged survival of cells (Jongpaiboonkit et al., 2008). Therefore, incorporating protease-sensitive sequences into these ECM matrices could expedite their degradation, addressing issues related to extended cell survival (Flora et al., 2019).

#### 4.1.1 Solid scaffold

One of the first reports on the modification of tissue culture polystyrene (TCPS) plates with ELR-inspired coatings as an alternative to conventional scaffold-free techniques appeared in 2008 (Janorkar et al., 2008). Janorkar et al. investigated the effect of ELR coatings on the viability and differentiation of primary rat hepatocytes. For this purpose, they employed neutral, negatively charged and positively charged ELR-based coatings. To create ELRs with positive or negative charges, a non-charged ELR, (VPGVG)_40_, was chemically conjugated to poly (ethylenimine) (PEI) and polyacrylic acid (PAA), respectively, by using carbodiimide chemistry. ELRs and ELR conjugates were adsorbed to TCPS and coated with primary rat hepatocytes. They demonstrated that neutral and negatively charged coatings supported cell-surface interactions, resulting in a cell monolayer within 48 h with increased secretion of liver-function-specific markers compared to non-coated surfaces. In contrast, positively charged coated surfaces promoted cell-cell cohesive interactions with the formation of cellular aggregates within 48 h. These aggregates subsequently formed three-dimensional spheroids with a diameter of 113 ± 6 µm within 72 h and with a higher-liver specific function than ELR-PAA surfaces (Janorkar et al., 2008).

The group continued their studies with ELR-PEI coatings, aiming to study the role of surface amination by using different molecular weights of PEI. ELRs were conjugated to PEI, with one having a molecular weight (M_w_) of 25,000 Da (ELR-PEI25000) and the other with a M_w_ of 800 Da (ELR-PEI800). The size of the human adipose-derived stem cells (hASCs) spheroids formed atop the ELR-PEI25,000 coatings appeared smaller compared to those formed atop the ELR-PEI800 coatings. The PEI with a higher M_w_ occupied more surface area, leading to more effective repulsion of cells. Consequently, this cell-PEI repulsion resulted in the formation of the smallest spheroids with an average diameter of approximately 50 μm. It was observed that the lowest aminated surface studied promoted a greater cell motility, stimulating the organization of smaller spheroids than those on highly conjugated ELR-PEI surfaces that prevent such organizations (Turner et al., 2014; Fitzgerald et al., 2020). The studies with ELR-PEI coatings continued due to their potential for cellular differentiation (Gurumurthy et al., 2017; Turner et al., 2017). In these works, hASCs cultured atop ELR-PEI formed 3D spheroids and exhibited superior osteogenic and adipose differentiation when supplemented with differentiation media, compared to the traditional 2D monolayer (Gurumurthy et al., 2017; Turner et al., 2017). ELR-PEI coatings were also proven to control the morphology of human mesenchymal stem cells (hMSCs) spheroids (Adams et al., 2014). ELR-PEI standardized hMSCs morphology due to the interaction of the polymer coating with the cell membrane.

In a different study, biofunctionality of ELRs was extended by incorporating integrin-binding sequences, such as the fibronectin-derived Arg-Gly-Asp (RGD), into the backbone of ELR (Lee et al., 2013). Jeon et al. studied how RGD-bearing ELRs affected the stimulation of fibroblasts and neuroblasts cells (Jeon et al., 2011), as well as the improvement of functional behavior and differentiation of neural cells (Jeon et al., 2012). Cell-adhesive ELRs are effective in modulating the spreading, proliferation, and differentiation of various types of mammalian cells. Lee et al., reported that this matrix promotes the clustering of β-cells into three-dimensional islet-like structures (pseudoislets) with high cell viability and higher insulin release compared to that of monolayer culture (Lee et al., 2013).

In summary, spheroids fabricated on ELR coatings avoid some drawbacks of other scaffold-free spheroid fabrication techniques, such as preventing dislodgement, loss in viability caused by shear stress, and control over spheroid kinetics. However, these anchorage-independent spheroids are comparatively fragile in culture and require precise handling to avoid disruption or loss. Thus, the scaffold-based approach utilizing hydrogels appears to 3D cell culture, involving anchorage-dependent spheroid formation (Shen et al., 2021) (Zhang et al., n. d.).

#### 4.1.2 Hydrogels

The utilization of scaffold-based approaches employing hydrogels offers physiological advantages to cultured cells, but the use of a hydrogel requires the recovery of embedded multicellular spheroids with intact architecture for further experimental manipulations (Hunt et al., 2021). For instance, the possibility to recover tumoroids from agarose gels was demonstrated by treating the hydrogels with agarase. However, the efficiency of this retrieval varied significantly depending on the stiffness of the gel matrix; while softer gels showed high reproducibility in the recovery process, stiffer matrices (0.25% agarose) yielded only a small percentage of successfully recovered 3D structures (Quarta et al., 2021). Commonly employed methods for removing Matrigel from embedded samples include Cell Recovery Solution and Dispase. Matrigel can be depolymerized using cell recovery solution at 4 °C without the need for enzymes, or by utilizing dispase, a neutral protease from *Bacillus polymyxa*, which digests Matrigel at physiological temperature (37 °C) without inducing biochemical reactions due to low temperatures (Abe et al., 2018). In this context, the temperature-responsive phase transition property of ELR could offer a straightforward approach for collecting embedded spheroids by simply lowering the temperature. This method avoids the use of enzymes, which can degrade extracellular domains of cell surfaces (Rana et al., 1994), eliminates the need for biochemical reactions triggered by very low temperatures (e.g., 4°C), and circumvents issues associated with stiffness characteristics. Consequently, scaffold-based approaches employing ELR hydrogels could overcome a significant obstacle in the retrieval of spheroids embedded in commonly used matrices (Kozlowski et al., 2021; Kozlowski et al., 2023).

ELRs have been used alone or combined with natural and synthetic polymers to produce hydrogel scaffolds (Caves et al., 2011; Camasão et al., 2020). One example was reported by Amruthwar et al., where they prepared a series of ELR-collagen composite scaffolds by varying the ratio of ELR ((VPGVG)_120_) to collagen. Mechanical testing experiments showed that ELR-collagen scaffolds exhibit superior mechanical attributes than pure collagen scaffolds, which usually suffer from poor mechanical properties and rapid degradation, thus influencing both cell morphology and differentiation potential (Amruthwar and Janorkar, 2013). In a different study, it was observed that chemical crosslinking of ELR-collagen matrices can affect hASC fate. Softer non-crosslinked scaffolds led to spread hASC morphologies, while stiffer crosslinked scaffolds constricted the spreading of hASCs, thus resulting in a spheroid morphology and promoting an enhanced adipogenic differentiation (Newman et al., 2020b). Highlighting the significance of hydrogel stiffness and hASC differentiation, ELR-collagen hydrogels induced osteogenesis and helped to control drug release when loaded with doxycycline and recombinant human bone morphogenetic protein-2 (rhBMP-2) (Pal et al., 2019).

A different work demonstrated that ELR-based hydrogels used for spheroid fabrication can be also decorated with growth factors to promote tissue regeneration, increasing local concentration and cell signaling events (Cai et al., 2014). Cai et al. developed a single-step, cell-compatible method to tether small growth-factor-mimetic peptides into ELR hydrogels. Human umbilical vein endothelial cell (HUVEC) spheroids were encapsulated within ELR hydrogels together with an angiogenic peptide (QK peptide). Soluble QK, when not tethered, diffuses away from the site of action and escapes from the hydrogel. In contrast, by tethering the QK peptide to the ELR hydrogel, concentrations of QK were sustained, resulting in prolonged angiogenic signaling and enhancing endothelial outgrowth. Overall, this work demonstrated that the use of ELR hydrogels for spheroids encapsulation fabrication enables precise tuning of matrix properties for effective 3D cell culture.

**FIGURE 3:**
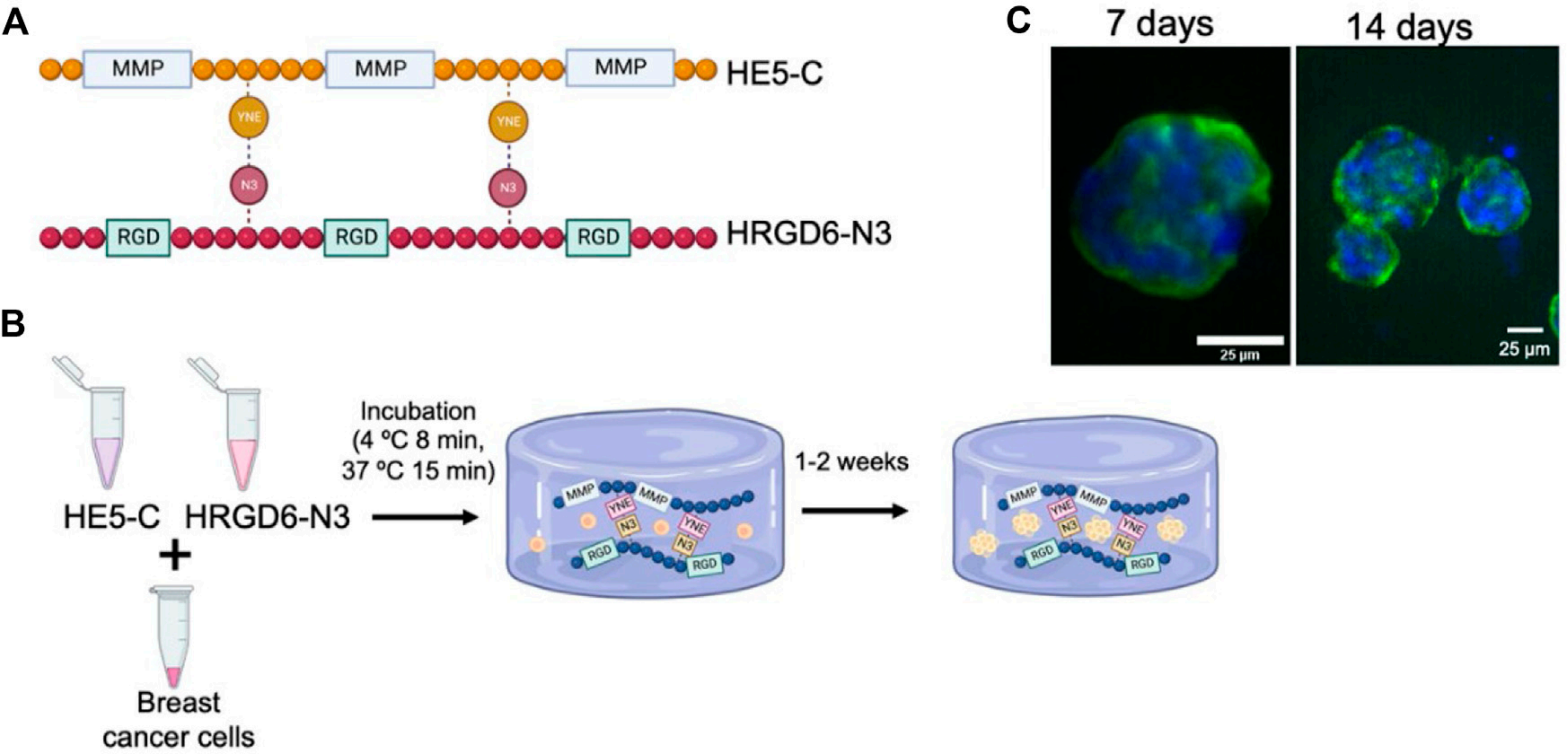
**(A)** Schematic representation of the ELR sequences used by Blanco-Fernández et al. for cancer cells spheroid formation. HRGD6 with RGD cell adhesion motif, and HE5 incorporating metalloproteinase-cleavage domains. **(B)** Procedure for generating the ELR cell-laden hydrogels: cells were combined with both ELRs (HE5-C and HRGD6-N3) and subjected to incubation at 4°C and 37°C to facilitate hydrogel formation. Hydrogels were kept under culture for up to 2 weeks. **(C)** Fluorescence images of the spheroids generated by MCF10A cancer cells in cell-laden ELR hydrogels after 7 and 14 days of incubation (green, cytoskeleton; blue: nuclei). (Scale bar: 25 µm). Reproduced with permission from (Blanco-Fernandez et al., 2022).

In the context of cancer research, recognizing the role of ECM in tumor progression has led to the development of biomaterials mimicking the tumor ECM for more accurate cancer models. Currently, 2D and animal models dominate cancer research, but they have limitations in replicating the tumor microenvironment. (Fong et al., 2016). To address this, 3D *in vitro* cancer models are gaining interest. In this case, scaffold-free platforms involve cancer cells forming spheroids, while scaffold-based systems anchor or encapsulate cancer cells in ECM-like biomaterials. (Micek et al., 2020). Recently, protein-engineered hydrogels have emerged as promising for cancer modeling due to their ability to replicate the mechanical and biochemical properties of ECM tumors. In this context, Blanco-Fernandez et al. used ELR hydrogels to recreate the breast cancer ECM for *in vitro* modeling. (Blanco-Fernandez et al., 2022). In this work, hydrogels were fabricated using two distinct ELRs: one featuring the RGD cell adhesion motif (HRGD6), and the other incorporating metalloproteinase-cleavage domains (HE5) (Figure 3A). HRGD6 was modified with azide groups, while HE5 was conjugated with cyclooctyne groups, leading to the formation of a crosslinked gel upon mixing (Figure 3B). These hydrogels supported optimal cell viability and proliferation for 1 week when breast epithelial and cancer cells were encapsulated. Different cell lines exhibited distinct behaviors, either forming spheroids (non-metastatic cell line (MCF7) and non-tumorogenic breast epithelial cells (MCF10A)) (Figure 3C) or forming cell networks (a triple-negative and metastatic cell line (MDA-MB-231)), with the expression of ECM proteins and high drug resistance against doxorubicin in all cases. These results evidence the potential of ELR hydrogels for developing breast cancer models to study drug resistance, cell invasion, and ECM secretion by cancer cells.

### 4.2 ELRs for organoid models

Organoids have gained significant attention for their potential applications in modeling tissue development, understanding diseases, and advancing personalized medicine, drug screening, and cell therapy (Hofer and Lutolf, 2021). They are also a promising tool to limit the need for animal experimentation. These scale-down models of organs can be generated for a variety of organs, such as the gut, stomach, kidney, liver, pancreas, mammary glands, prostate, upper and lower airways, thyroid, retina, and brain (Takasato et al., 2015; McCracken et al., 2017; Workman et al., 2017; Hu et al., 2018).

Despite their potential, the translation of organoids to real-life applications remains a significant challenge. One of the biggest obstacles to clinical translation is the ill-defined growth environments of organoids, leading to a high variability and heterogeneity in cellular composition and in the resulting organoid phenotype (Hofer and Lutolf, 2021). Relying only on cell-intrinsic self-organization limits external control over fate and morphogenesis and often lacks stromal, vascular and immunological components (Zhao et al., 2022). Therefore, it is crucial to design environments that not only support stem cell maintenance but also enable precise spatiotemporal modulation of bioactive cues to guide organoid growth leading to a best understanding of organogenesis.

Examples of this approach include the application and combination of hydrogel chemistries and organ-on-a-chip technology. This integration aims to better mimic the stem cell niche by delivering and presenting *in vivo*-based biochemical cues, as well as incorporating biophysical and topological parameters often lacking in traditional culture systems (Kratochvil et al., 2019; Hofer and Lutolf, 2021; Zhao et al., 2022). Based on these insights, engineered matrices offer the potential for enhanced control over supplied morphogenic signals in both space and time, allowing precise control over stem cell decisions during organoid development (Hofer and Lutolf, 2021).

**FIGURE 4:**
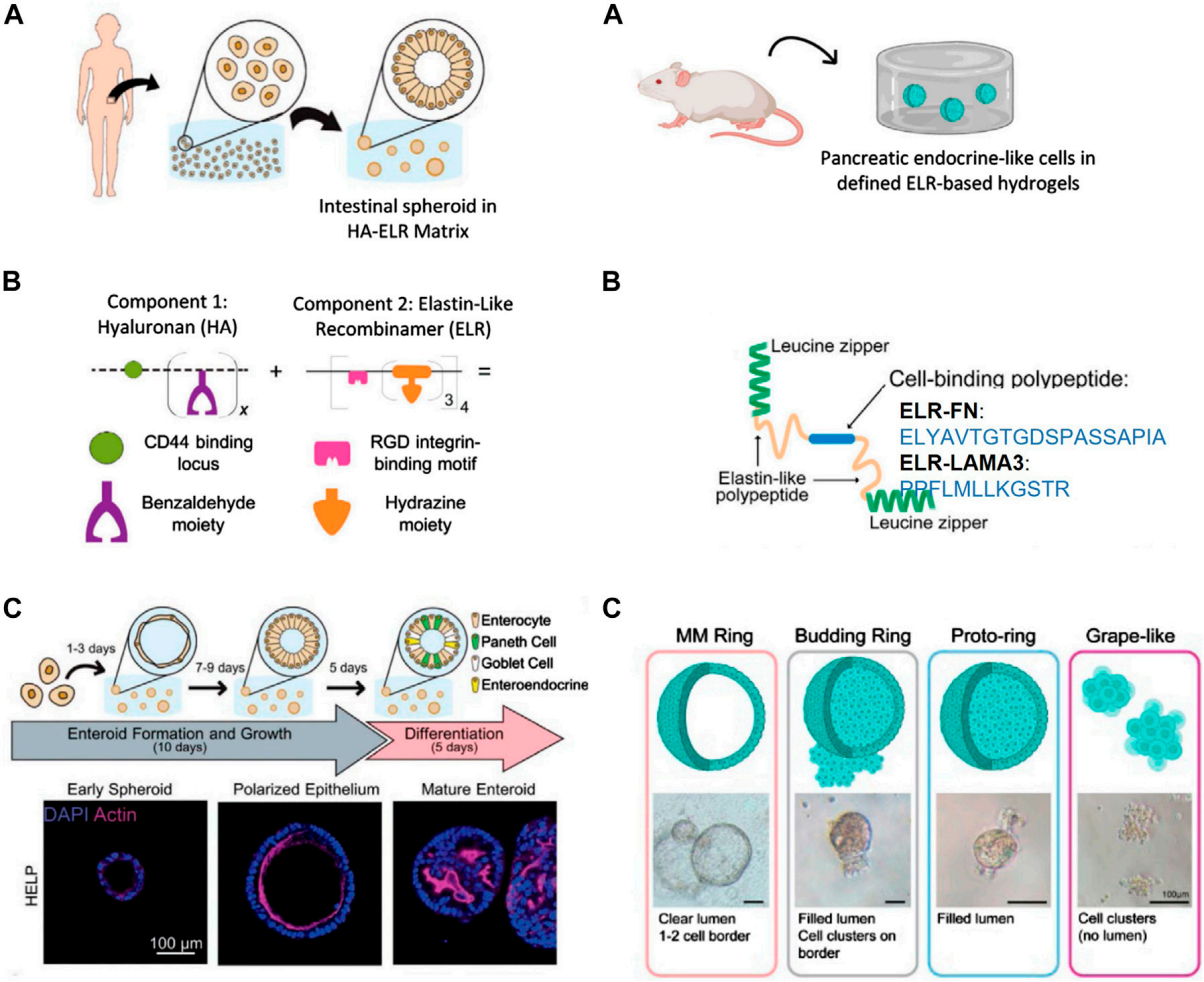
Representative images of intestinal (left panel) and pancreatic (right panel) organoids in defined protein-based hydrogels. **(A)** Source of progenitor cells. Left: intestinal tissue biopsies from human patients for intestinal organoids. Right: pancreatic progenitor cells from 8-day-old -mice for pancreatic organoids. **(B)** Left: schematic of HA-ELR matrix, which is composed of benzaldehyde-modified hyaluronan (HA) and hydrazine-modified elastin-like recombinamer (ELR) leading to the formation of chemical cross-linked gel (hydrazine bonds). Right: schematic drawings of ELR-FN and ELR-LAMA3 proteins, which contain the following main elements: 1) leucine zipper (green) which forms bundles in solution, leading to a physically cross-linked gel, 2) elastin-like recombinamers (yellow), and 3) the cell binding peptide (blue; either the RGD peptide, or laminin three alpha mimetic) **(C)** Left: differentiation experiment and progression of organois from early enteroid to polarized enteroid to differentiated organoid. Left: representative photos of various colony types. Reproduced with permission from (Hunt et al., 2021; Kozlowski et al., 2023).

In the pursuit of organoids as models for organs, numerous researchers seek to address diseases that impact a significant portion of the population, such as type 1 diabetes (Xu et al., 2018). This condition leads to the destruction of pancreatic beta cells, with the transplantation of cadaveric human islets emerging as a promising alternative (Ames et al., 2000; Shapiro et al., 2016). However, the clinical demand surpasses the available supplies (Walker et al., 2022). For this reason, several research groups have explored the production of functional beta-like cells from pluripotent stem cells (Pagliuca et al., n. d.; Walker et al., 2022). Many of these methods rely on the use of Matrigel, but its undefined composition makes it difficult to understand which factors are responsible for differentiation, as well as tuning of its mechanical properties, that are critical in stem cell differentiation (Goldstein et al., 2011). A recent breakthrough in the ability of ELR to support the survival and growth of primary endocrine cells and endocrine progenitor cells in a 3D space was reported by Kozlowski et al. The group introduced a novel method for culturing pancreatic endocrine-like cells without the use of Matrigel (Kozlowski et al., 2023). ELRs containing cell-binding ECM peptides derived from fibronectin (i.e., ELR-FN) (Nakamura et al., 2022) or laminin alpha 3 (i.e., ELR-LAMA3) (Tjin et al., 2014), essential for pancreatic endocrine functions, were engineered. For this study, the researchers chose the 158A mutant to match the stiffness of the Matrigel-methylcellulose medium as closely as possible. They found that 2% solutions of ELR-FN or ELR-LAMA3 form semisolid matrices with elastic moduli comparable to the ideal modulus for human forebrain and human and mouse intestine, and to that of the Matrigel-methylcelluose medium, which is known to support the growth of primary murine pancreatic ductal progenitor cells. (Jiang et al., 2002). These ELRs are flanked by leucine zipper domains from rat cartilage oligomeric matrix protein, forming bundles in solution, leading to the formation of a physically cross-linked hydrogel (Figure 4B right). Both protein hydrogels supported the survival and growth of primary endocrine cells and endocrine progenitor cells in 3D space, compared to the control culture containing Matrigel, which favors ductal cell formation, and to DMEM-F12 medium, where cells remain in suspension and re-aggregate. Pancreatic progenitor cells cultured in ELR-FN or ELR-LAMA3 exhibited several types of colonies termed “grape-like”, “budding ring” and “proto-ring”, more endocrine in nature. However, Matrigel-methylcellulose medium employed in recent studies by this group supported the growth of “MM Ring” colonies, more duct-like and proliferative (Figures 4A,C right). Thus, their study provides a proof-of-concept cell culture matrix for endocrine cell differentiation in young mice without Matrigel.

Despite the utilization of various complex materials in the development of 3D pancreatic organoids (i.e., Matrigel (Huch et al., 2013), decellularized intestinal tissues (Giobbe et al., 2019) and decellularized pancreatic tissues (Sackett et al., 2018)), these results are consistent with the possibility of growing various progenitor cell types in 3D culture without Matrigel. Furthermore, the tunability of the mechanical and chemical properties of the protein elastin-like hydrogels described makes them valuable for conducting mechanistic studies on endocrine cell differentiation.

In an effort to overcome the limited tunability and reproducibility associated with Matrigel (Kozlowski et al., 2021), synthetic matrices have been developed to support the formation of induced pluripotent stem cell (iPSC)-derived intestinal organoids (Gjorevski et al., 2016; Cruz-Acuña et al., 2017; Hushka et al., 2020). Mohimani et al. utilized PEG as a synthetic polymer but faced challenges due to its interaction with the immune system and antibody formation (Mohimani et al., 2017).

Addresing these limitations, Heilshorn’s team introduced a tunable and fully defined matrix termed based on hyaluronan ELR composite protein (HA-ELR). (Hunt et al., 2021). This matrix enables the formation, growth, passaging, and differentiation of human intestinal organoids from dissociated patient-derived intestinal cells (Figure 4A left) Elastin constitutes a significant portion of the intestinal ECM, while HA serves as a crucial glycosaminoglycan supporting normal intestinal growth and interacting with the CD44 receptor responsible for maintaining and proliferating intestinal stem cells. This rationale led them to develop a reproducible hydrogel incorporating these two components. HA-ELR gels were formed through straightworward bioconjugations reactions by modifying hydrazine-bearing ELRs and benzaldehyde-bearing HA, thus leading to the formation of dynamic covalently cross-linked hydrogels (Figure 4B left). Human, patient-derived enteroids embedded within HA-ELR exhibited *de novo* spheroid formation within 3 days. This contrasts with ELR matrices, which were unable to support enteroid formation, indicating the necessity of HA for robust enteroid formation. Additionally, enteroids formed in HA-ELR exhibited proper intestinal epithelial polarity similar to decellularized matrix derived from Engelbreth-Holm-Swarm mouse sarcoma (e.g., Matrigel). HA-ELR matrices can be enzymatically degraded using elastase and hyaluronidase, facilitating enteroid dissociation into single cells for subsequent passages. Successful new enteroid formation for up to 12 passages without visible change in morphology was achieved. To assess whether HA-ELR could support the differentiation of enteroids, cells embedded in HA-ELR were allowed to form enteroids for 10 days before differentiation into mature organoids over 5 days. This resulted in the formation of undulating lumens, indicative of mature intestinal cell subtypes (Figure 4C left). The study specified matrix stiffness, stress relaxation rate, and integrin-ligand concentration independently and quantitatively, enabling fundamental studies of organoid-matrix interactions and potential patient-specific optimization. This work shows that HA-ELR provides a promising 3D *in vitro* model for understanding intestinal development and enteric disease, devoid of animal-derived products or synthetic materials with clinical translation issues. It supports enteroid growth and differentiation akin to animal-derived matrices, offering a reproducible, biodegradable, minimal matrix for potential clinical applications.

Although 3D organoid tissue models have an invaluable valor to study several biological processes, several techniques such as immunostaining, mechanosensing, mechanotransduction and optical strategies have been refined over the years for application in 2D cultures (Yousafzai and Hammer, 2023). For this reason, current limitations in processing organoids limit the capacity for higher-throughput analysis. Adapting these techniques to 3D culture models will enhance the advancement of our understanding of cellular behavior and facilitate organoids processing. In this context, Kaplan’s team fabricated smart material hydrogel transfer devices utilizing stimuli-responsive silk-elastin-like recombinamers (SELRs) for use in organoid histological processing (Parker et al., 2020). The procedure involves transferring organoids to SELR hydrogel, followed by subjecting the material to an increase of the temperature (65°C) to induce hydrogel contraction, securing the organoids and enabling the creation of multisample constructs, and allowing placement on a microscope slide. Histological processing (i.e., hematoxylin and eosin staining) and immunostaining of these organoids using SELR hydrogels demonstrated the maintenance of cerebral organoid features compared to controls without the hydrogel carrier system. The utility of SELR devices stems from their optimal design, which not only maximizes material surface area but also enhances throughput and ensures the proper fit of organoids. The future investigation of SELR constructs holds potential for the development of materials on a broader scale, particularly for applications such as clinical diagnostic screening of patient tissue samples.

### 4.3 ELRs for organ-on-a-chip models

Organs-on-a-chip (OOAC) are sophisticated systems that incorporate engineered or natural miniature tissues cultivated within microfluidic chips, to replicate the specific functions of living human organs (Leung et al., 2022). These systems offer a 3D environment responsive to mechanical, electrical, and chemical stimuli in small samples, thereby reducing the need for substantial amounts of reagents or extended analysis times (Osório et al., 2021). OOAC represents an animal-free technology with predictive capabilities, ultimately reducing costs and time associated with pre-clinical testing.

Examples of emerging OOAC technologies include heart-on-a-chip, lung-on-a-chip, kidney-on-a-chip, bone-on-a-chip, liver-on-a-chip, and skin-on-a-chip where most of them are based on poly(dimethylsiloxane) (PDMS) chip with a Matrigel matrix.(Leung et al., 2022) However, the materials currently employed in OOAC research face challenges that can compromise the reliability of these systems as pre-clinical models (Danku et al., 2022). A comprehensive review by Luana A. Osório et al. (Osório et al., 2021) discusses the various biomaterials employed in OOAC systems and their associated limitations. ELRs proven properties have a great potential of application to solve the existint limitations of the materials commonly used in OOAC technologies. For instance, PDMS, biocompatible material extensively employed in biofabrication due to its excellent transparency and elasticity, exhibits poor chemical resistance, absorbing certain organic compounds, drugs, and biomolecules (Koyilot et al., 2022). While PDMS is widely used in lung-on-a-chip owing to its flexibility, it remains a non-degradable material that fails to contribute to the formation of a natural ECM (Huh, 2015). The biodegradable properties of the ELRs could help to solve this problem. Another material commonly used is Matrigel, a biocompatible material with similar mechanical properties to natural ECM and used in liver-on-a-chip. However, Matrigel introduces variability in experimental results due to batch-to-batch differences (Jang et al., 2019). Conversely, the recombinant design and bioproduction of ELR provides batch-to-batch consistency and extreme control over composition (Walker, 2009). Collagen, one of the major components of ECM, offers favorable permeability, biocompatibility, and enzymatic degradation, enabling cellular remodeling of the ECM gel. Although widely used in cardiac, hepatic and gut-on-a-chip, collagen lacks mechanical strength and structural stability when hydrated (Arlk et al., 2021). ELR-collagen scaffolds have demonstrated superior mechanical attributes compared to pure collagen scaffolds, thereby influencing cellular morphology and differentiation potential (Amruthwar et al., 2013). Similarly, fibrin, another biocompatible and biodegradable material, allows gel modeling at room temperature but exhibits weak mechanical properties. Fibrin hydrogels have been extensively studied for tissue engineering, but they show rapid degradation and contraction over time and low mechanical properties. To achieve better mechanical properties and elasticity, Stojic et al. incorporated ELRs network into plasma-derived fibrin hydrogels (Stojic et al., 2021). Their results demonstrated that incorporation of ELRs significantly increased mechanical properties, as determined in tension and oscillatory shear, when ELR content is equal or higher than 3%. This superior behavior is also reflected in terms of shrinkage, both in the absence and presence of cells, where an increase of scaffold stability was observed with the increase of ELR content. In contrast, synthetic biomaterials offer regulable mechanical properties but necessitate evaluation of immune responses due to potential cytotoxic effects. ELRs are non-immunogenic (Ibáñez-Fonseca et al., 2020) and the biocompatibility of ELR-based hydrogels formed via physical or chemical crosslinking has been established (Ibáñez-Fonseca et al., 2018).

These challenges, alongside the advantages offered by OOAC in pre-clinical settings, have spurred rapid developments in biomaterials and manufacturing techniques. However, each biomaterial presents limitations in achieving the optimal scaffold for these systems. Consequently, there is a concerted effort towards the development of 3D scaffolds capable of meeting multiple criteria, including biocompatibility, biodegradability, pore size, and suitable mechanical properties. The significant advantages of ELRs highlighted throughout this section underscore the potential of ELRs as a promising material for future applications in this field. These advantages, as discussed, not only showcase the potential of ELRs to address the limitations associated with current biomaterials, but also strongly advocate for their integration into OOAC systems.

### 4.4 3D bioprinting of complex *in vitro* models

3D bioprinting, arising from additive manufacturing, involves the automated and precise deposition of cells, biomaterials, and bioactive molecules, collectively forming a “bioink.” It addresses the limitations of conventional 2D platforms by enabling the creation of scaffolds with tailored structural and biochemical properties (Jain et al., 2022; Lam et al., 2023; Gold et al., 2019; Matai et al., 2020; Ahadian and Khademhosseini, 2018; Agarwal et al., 2020; Noroozi et al., 2023; Aazmi et al., 2024). This capability is pivotal for generating *in vitro* models that accurately mimic the intricate features of native tissues, facilitating regenerative medical endeavors (Nam et al., 2015; Langhans, 2018; Bédard et al., 2020; Hwang et al., 2021). Recent examples have highlighted the potential of 3D bioprinting in creating *in vitro* models of human tissues and diseases, including notable applications such as: accurate modeling of the complex tumor microenvironments (Kort-Mascort et al., 2023; Mu et al., 2023; Zhou et al., 2023); physiologically relevant models for the entire respiratory tract (Moura et al., 2023); replication of native skin features for wound healing studies (Norahan et al., 2023); engineered cardiac tissues such as *in vitro* cardiac models and vascular channels (Lu et al., 2023); or normal and disease hepatic tissue models (Sun et al., 2023).

Various commonly used 3D bioprinting methods such as extrusion, inkjet, and laser-assisted methods contribute to the precise control of spatial cell distribution and the surrounding microenvironment (Filipa Duarte Campos et al., 2016) (Derakhshanfar et al., n. d.) (Li et al., 2020). Furthermore, innovative approaches such as liquid-in-liquid printing (Luo et al., 2019) (Chen et al., n. d.), 3D Embedded Printing (Miller et al., 2012; Kyle and Jessop, 2017; Aazmi et al., 2022; Wu et al., 2022a; Wu et al., 2022b; Li et al., 2022; Budharaju et al., 2024), Freeform Reversible Embedding of Suspended Hydrogels (FRESH) printing (Hinton et al., 2015; Lee et al., 2019), Suspended Layer Additive Manufacturing (SLAM) (Grigoryan et al., 2019; Noor et al., 2019; Senior et al., 2019; Shapira et al., 2020), and light-based vat-polymerization techniques such as Volumetric bioprinting (VAM) (Bernal et al., 2019; 2022; Größbacher et al., 2023; Levato et al., 2023; Ribezzi et al., 2023), have emerged as a potential tool to print soft materials, which is particularly intricate (Puertas-Bartolomé et al., 2020; Budharaju et al., 2024).

However, despite significant advances in bioprinting techniques in the last years, bioprinting faces a significant challenge in the limited repertoire of suitable bioinks compared to the diverse biomaterials used in traditional tissue engineering scaffolds (Levato et al., 2020) (Gungor-Ozkerim et al., n. d.; Hull et al., 2021). Various factors contribute to this limitation, including biocompatibility, rheological properties, crosslinking and gelation characteristics, and mechanical properties. The selection of suitable bioinks is critical for successful bioprinting. On the one side, a bioink must not only be compatible with the printing process but also create a conducive environment for cell survival, growth, and differentiation, as well as specific cues to meet (Ashammakhi et al., 2019; Urciuolo et al., 2023) (Ihalainen et al., n. d.) the unique needs of different cell types, fostering a more accurate representation of the native tissue environment. On the other hand, the bioink should have a specific viscosity and shear-thinning behavior to facilitate extrusion through the printing nozzle, support the layer-by-layer construction, and maintain shape fidelity and structural integrity of the printed construct (Gungor-Ozkerim et al., n. d.; Malda et al., 2013) (Gungor-Ozkerim et al., n. d.; Malda et al., 2013). Additionally, the mechanical properties of bioinks are crucial for mimicking native tissue characteristics (Mandrycky et al., 2016).

Researchers are actively addressing the challenge of the restricted current range of bioinks by exploring novel biomaterials and refining existing formulations to expand the bioink toolkit (Gungor-Ozkerim et al., n. d.) (Gungor-Ozkerim et al., n. d.; Hull et al., 2021) and enable the creation of more complex and functional tissues. Hydrogels are currently the most versatile biomaterials used in bioink formulations. closely resembling the ECM mechanical properties, composition, and achitecture (Gungor-Ozkerim et al., 2018; Valot et al., n. d.; Tibbitt and Anseth, 2009; Gopinathan and Noh, 2018; Li et al., 2020). (Chemistry and 2020, n. d.). Naturally derived bioinks such as alginate, collagen, fibrin, gelatin, silk fibroin, hyaluronic acid, and decellularized extracellular matrix have been often used for the preparation of bioinks. They offer an environment comparable to the extracellular matrix, and essential properties like cell adhesion and proliferation (Gopinathan and Noh, 2018; Li et al., 2020). However, these materials often face challenges such as low batch-to-batch reproducibility due to their natural origin, and relatively low mechanical properties and structural drawbacks (Fedorovich et al., 2007; Gasperini et al., 2014; Ahadian and Khademhosseini, 2018). Furthermore, polysaccharides such as alginate and hyaluronic acid have yet to be functionalized to allow cell adhesion and proteolytic degradation. Bioinks based on synthetic materials like PLGA, pluronic acid, PEG, PLA, and PCL have also been commonly employed due to their superior printability (Gopinathan and Noh, 2018; Li et al., 2020). However, these synthetics struggle to provide an optimal cell environment and must be modified to enable functions such as cell adhesion, proliferation, or proteolytic degradation (Smith et al., 2004; Censi et al., 2011; Chang et al., 2011; Zhang C. et al., 2016). Thus, a potential breakthrough lies in the development of biomimetic inks, merging the biological advantages of natural components with the design flexibility of synthetic polymers.

#### 4.4.1 ELRs as bioinks for 3D bioprinted models

ELRs have emerged in the last years as a promising category of bioinks that address the inherent limitations of current materials (Salinas-Fernández et al., 2020; Dai et al., 2021; Hull et al., 2021). This approach seeks to create a material that meets the diverse parameters determining structural, mechanical, and biological behavior for 3D bioprinting applications. These materials have adjustable initial stiffness (by varying the final concentration of ELR or crosslinker); can be engineered for controlled metalloprotease degradation facilitating local matrix remodeling and enable sustained cell proliferation over an extended period; are suitable for controlling encapsulated cell phenotype and stemness; and exhibit thermo-responsiveness (Chung et al., n. d.; Madl et al., n. d.). This smart behavior of reversible phase transition or self-assembly in response to the temperature can be leveraged for the deposition of elastin-like materials into 3D architectural matrices, offering a versatile and controllable approach in the preparation of bioinks. Groundbreaking studies, exemplified by those outlined in this Section, demonstrate the feasibility of achieving this goal.

**FIGURE 5:**
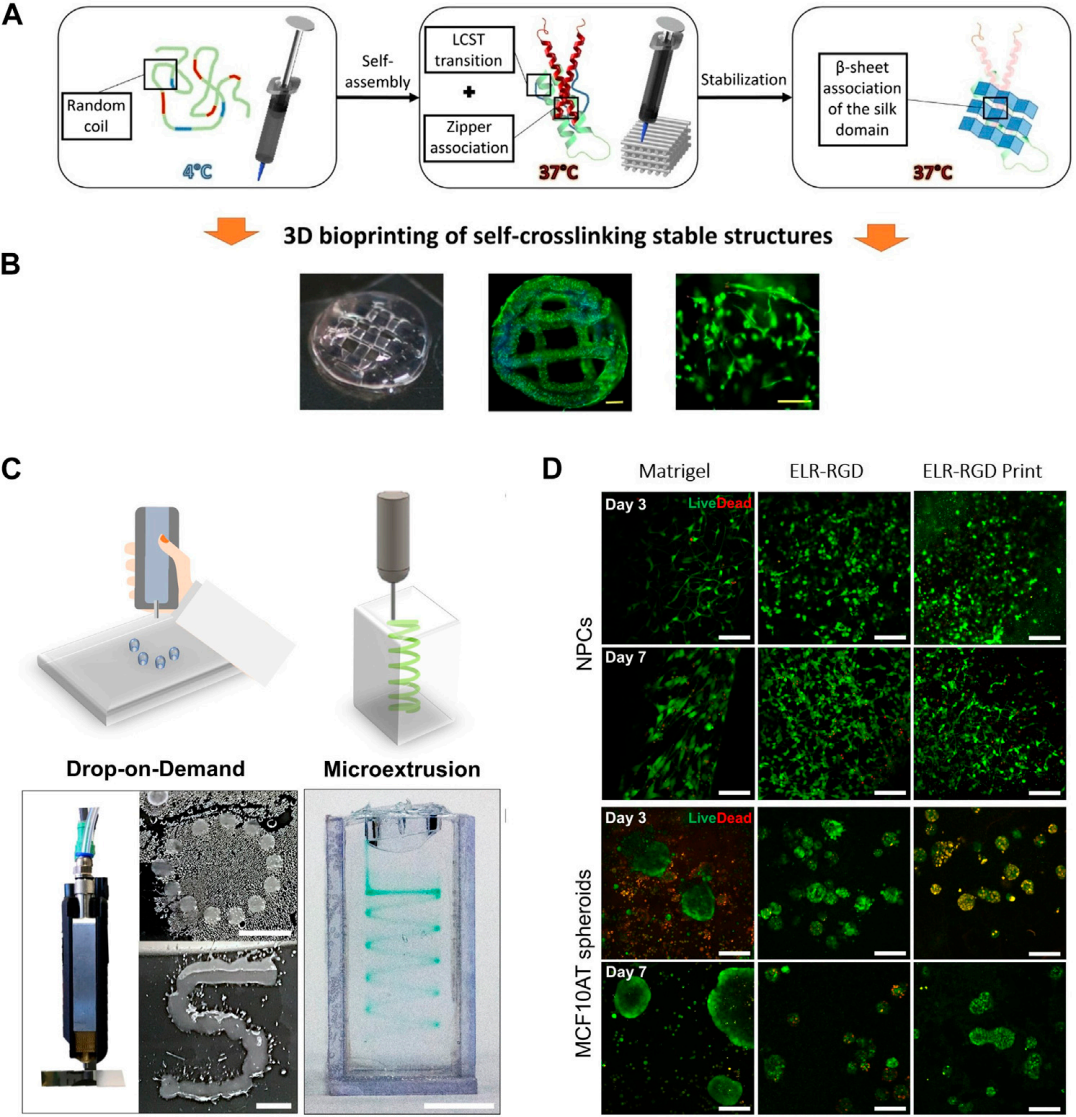
**(A)** Graphical scheme of the three sequential gelation steps of the ELR-based bioink developed by Salinas-Fernández et al. (Salinas-Fernández et al., 2020), and **(B)** microscope images of the printed scaffolds loaded with HFF-1 (Dapi/Phalloidin staining), and a magnified zone into the middle (Live/Dead staining) after 21 days of culture. Scale bar for DAPI/phalloidin: 500 μm; for live/dead: 200 μm. Reproduced with the permission from (Salinas-Fernández et al., 2020). **(C)** Qualitative drop-on-demand (DoD) printability tests as single drops into circular and S shapes, and microextrusion printability test in a spiral shape within a Pluronic bath of ELR-based bioink. Reproduced with permission from (Duarte Campos et al., 2020) Scale bar represents 5 mm. **(D)** Live/Dead staining of murine neural progenitor cells (NPCs) and human premalignant breast epithelial cell spheroids (MCF10ATs) on days 3 and 7 within the ELR-based bioink formulations developed by Duarte Campos et al. and a Matrigel control (3 wt% ELR-RGD (not bioprinted), and 3 wt% ELR-RGD) after DoD bioprinting. Scale bars represent 100 μm. Reproduced with permission from (Duarte Campos et al., 2020).

In the pursuit of designing a bioink with both printability and stability, our team was a pioneer in creating advanced printed scaffolds by 3D extrusion bioprinting based on ELRs (Salinas-Fernández et al., 2020). The study aimed to induce a sol-gel transition in ELR through three sequential gelation steps (Figure 5A). Firstly, a thermally induced fast gelation, relying on hydrophobic interactions, due to the presence of the amphiphilic tetrablock recombinamer (EI) in the ELR (Fernández-Colino et al., 2014). This rapid gelation right upon printing facilitates shape fidelity and avoids material collapse. Secondly, a stabilization stage was introduced by incorporating in the ELR a leucine zipper domain from the dimerization domain of the hepatic leukemia factor (Vinson et al., 2002; Fernández-Colino et al., 2015). This step ensures structural stability during the printing process. A third step for stabilizing the printed structures involves a gradual process of conformational changes. This is achieved by introducing a silk-like peptides (a consensus *Bombyx mori* fibroin peptide, GAGAGS) within the ELR backbone, combining the characteristics of elastin and silk. The inherent ability of this peptide to form β-sheets results in a gradual and long-term tightening of the printed material. The sophisticated molecular design of the ELR-bioink resulted in an extrudable bioink with high printability and suitable mechanical properties, thanks to the three-stage gelation process. This ELR-derived bioink effectively addresses key limitations seen in other natural and synthetic bioinks presenting batch-to-batch consistency, high mechanical stability, and shape fidelity. Additionally, the bioink provides an optimal extracellular environment for cell growth and proliferation due to the incorporation of a terminal bioactive block containing the integrin-dependent cell-adhesion RGD tripeptide (Figure 5B). This research marks a significant step in showcasing the applicability of elastin-like materials for the preparation of bioinks for tissue engineering and opens avenues for future customization by incorporating specific sequences within the amino acid chain to introduce innovative properties and bioactivities for the development of personalized tissue bioinks.

The development of self-assembling bioinks presents an exciting prospect for biofabrication. In their recent work, Mata´s team takes advantage of the molecular self-assembly of the ELRs for the preparation of a self-assembling bioink (Chen and Zou, 2019)^.^ The bioink system, composed of an ELR and graphene oxide (GO), showcased the potential of this system in liquid-in-liquid bioprinting for the creation of perfusable fluidic devices (Wu Y. et al., 2021). In this study, the authors performed a comprehensive standardization to optimize printing parameters and establish a reproducible process. Capillary-like structures with a diverse range of structural and biofunctional properties not typically achievable with most bioinks were achieved, such as fine resolutions (luminal diameters down to ∼10 μm and wall thicknesses of ∼2 μm), tunable permeability (both with and without cells), and permeability gradients within a single structure. This work illustrates the potential of leveraging the self-assembly property of ELRs for the preparation of self-asssembling bioinks, which in combination with liquid-in-liquid bioprinting technique, represents a significant advancement in fabricating biomimetic structures with physiological relevance.

In a different study, Lecomandeux´s team describes the use of ELRs for the preparation of a novel photocrosslinkable bioink for inkjet bioprinting (Dai et al., 2021). In this case, the group employed chemoselective postmodification reactions to selectively modify specific residues in the recombinant ELRs (Kramer et al., 2015; Petitdemange et al., 2017). This approach aimed to confer new functionalities and properties without the need for time-consuming molecular cloning steps. Specifically, they selected an ELR containing valine and methionine residues, and incorporated acrylate moieties into the methionine residues for chemical cross-linking via photopolymerization. Additionally, to facilitate cell adhesion, they combined the ELR with Collagen I, or integrated into the ELR the peptide sequence Gly-Arg-Gly-Asp-Ser (GRGDS). This GRGDS sequence includes the RGD sequence, which mediates interactions between the matrix and cell membrane receptors (Bellis, 2011). It is noteworthy that ELRs lost their thermosensitive property after undergoing modification. The incorporation of biocompatible cellulose nanofibers (CNF) to the bioink formulation (a strategy that has often been used in bioink compositions) was necessary for achieving the printing of liquid elastin-like protein bioinks with good resolution, contributing to the appropriate bioink consistency during printing and likely responsible for the shear-thinning behavior of the bioinks (Athukoralalage et al., 2019). The selection of inkjet bioprinting for scaffold construction was based on its reported advantages, such as relatively higher cell viability compared to extrusion bioprinting (Murphy and Atala, 2014; Valot et al., 2019). However, it requires materials with shear-thinning properties to function effectively. ELR-based bioinks demonstrated shear thinning behavior and good printability using this technique. The ELR-based bioinks exhibited a gel-like structure, providing effective support for cells before the printing process, preventing cell sedimentation. The resulting ELR-based printed structures demonstrated good resolution, stability and excellent mechanical properties, including stiffness and elasticity. Biocompatibility studies revealed that the designed ELR bioink alone lacked the capability to promote cell adhesion, while proved to be biocompatible, supporting adhesion and viability of dermal normal human fibroblasts (NHF) in the presence of collagen or the GRGDS peptide. Additionally, immunofluorescence studies of printed structures using these bioinks containing normal human fibroblast cells revealed the expression of specific ECM protein dermal markers such as pro-collagen I, elastin, fibrillin, and fibronectin. These findings collectively demonstrate that ELRs are a versatile and tuneable material that allows the preparation of customizable binks for 3D bioprinting, showcasing its potential for developing intricately designed and biocompatible constructs.

The Heilshorn team is also actively exploring the application of ELRs in the formulation of novel bioinks. Their focus lies on the development of new crosslinking strategies to overcome existing limitations, and enhance the efficacy of the bioprinting process. They highlight the use of biorthogonal chemistry as a crosslinking approach that not only expands the range of available bioinks, but also prioritizes cell compatibility and viability in the bioprinting process (Madl et al., 2016; Hull et al., 2021). Bioorthogonal chemistries facilitate the fast covalent reaction between two complementary functional groups (Madl et al., 2016; Dicker et al., 2018). These reactions offer significant advantages for bioprinting because they are chemically specific, do not react with biologically relevant functional groups, do not generate harmful byproducts, and occur rapidly at room temperature without external triggers (Madl et al., 2018). Specifically, the strain-promoted azide-alkyne cycloaddition (SPAAC) reaction between azides and bicyclononynes (BCN) has been commonly explored in previously reported works. (Marsico et al., 2021; González-Pérez et al., 2022). SPAAC chemistry crosslinking is a water-stable and copper-free click-chemistry that exhibits suitable reaction kinetics for homogeneous cell encapsulation. The group has employed this bioorthogonal crosslinking mechanism to develop a universal bioink approach for freeform bioprinting of diverse cell types and a variety of polymers. This family of materials is termed UNIversal, Orthogonal Network (UNION) bioinks. The UNION bioinks offer a versatile toolkit that can be tailored for specific biological applications thanks to the specificity of the crosslinking strategy. The preparation of UNION bioinks involves grafting one of the bioorthogonal chemical groups onto the polymer backbone before mixing with cells. The UNION bioink is extruded into a bath containing the complementary bioorthogonal group as a crosslinking molecule. The crosslinking molecule diffuses into the printed structure, initiating the spontaneous crosslinking reactions and enhancing the stability of the final structure. After liquefying the gel support bath, the printed structure is covalently crosslinked and can be easily extracted. This bioprinting method is compatible with any water-soluble polymer, non-cytotoxic, susceptible to conjugation chemistry with bioorthogonal functional groups, and extrudable. To showcase the versatility of this crosslinking approach, gelatin, hyaluronic acid, ELR, and polyethylene glycol (PEG) were tested. The bioink formulations required a relatively extended crosslinking time (1–4 h), raising concerns about cell viability and resolution. However, they demonstrated that encapsulated human corneal mesenchymal stromal cells (c-MSCs) into UNION bioinks of these materials presented high viability values (>85%) after the printing process, including crosslinking. Furtheremore, UNION strategy enabled the creation of cohesive structures from multiple materials with varied polymer compositions, thereby enhancing the potential for complex *in vivo* mimics and offering extensive customization possibilities in bioprinting.

In another study, Heilshorn´s team addressed another key limitation in bioprinting, which is the need for vascularization in printed constructs. As tissues grow beyond a certain thickness, the lack of a vascular network can lead to inadequate nutrient diffusion, compromising the viability and functionality of the innermost cells. Thus, creating a functional vascular network within printed tissues is essential for ensuring an adequate supply of nutrients and oxygen to cells throughout the structure (Tavazoie et al., 2008; Zhang Y. S. et al., 2016; Curtin et al., 2018), and different strategies such as microfluidic systems (Cochrane et al., 2019) to promote the formation of blood vessels within the printed constructs are being explored. In this recent study, Heilshorn et al. explore the use of ELR hydrogels as bioinks for the construction of 3D tissue models on microfluidic chips containing vascular-like channels (Duarte Campos et al., 2020). The ELR-based hydrogels selected incorporate the cell-adhesive RGD peptide. Drop-on-Demand (DoD) technique was selected for this investigation due to its reported cell-friendly and freeform nature compared to other methods (Blaeser et al., n. d.), and printability using a handheld bioprinting device was assessed (Figure 5C). The droplet size and weight showed to be affected by ELR-RGD concentration, printing pressure, and printing distance. Printability using microextrusion was also tested demonstrating the successful creation of intricate and freeform 3D geometries with high definition (Figure 5C). The viability of neural progenitor cells and cancer spheroids after the bioprinting process was assessed, revealing encouraging outcomes after a 7-day culture period (Figure 5D), and a sustained viability for up to 14 days, with observable spreading of neural progenitor cells. Finally, the authors utilized custom-designed on-chip platforms featuring vascular-like channels for integrated 3D bioprinting. They bioprint the ELR-based bioink containing human induced pluripotent stem cell (hiPSC)-derived NPCs onto a device with vascular-like, endothelialized channels as a proof of concept. Over a 5-day culture period, both cell types, NPCs and human umbilical vein endothelial cells (HUVECs), remained viable and maintained NPC stemness. The experiment demonstrated the feasibility of a synergistic approach involving bioprinting and on-chip technologies to achieve physiologically relevant geometric patterning in cocultures of NPCs and HUVECs.

## 5 Conclusion and future trends

*In vitro* models play a key role in understanding specific biological processes or functions, which can help in the prediction of human toxicological and pathophysiological responses, and offer valuable complements and potential substitutes for traditional animal testing. Despite significant progress, it remains difficult to accurately reproduce the complexity of native biological structures or tissues, with the limited availability of suitable biomaterials being a notable obstacle. Future trends in in vitro model development will encompass key points in the areas of biofabrication and biomaterial customization, which is crucial to recapitulate the critical functions of each cellular or tissue model. In this regard, ELRs emerge as versatile tools in this customization, with their unique properties such as phase transition behavior, tunable biological properties, viscoelasticity, and easy processability.

In terms of molecular models of IDPs, ELRs are excellent candidates for studying intracellular molecular crowding environments and understanding the mechanisms that control the phase transitions of complex IDPs, that regulate the formation of biomolecular condensates or membraneless organelles or the condensation of proteins in the ECM during growth stages. Given their sequence versatility and the possibility of transfecting them in cells, ELRs position as a potential synthetic biology tool to fabricate synthetic condensates for metabolic and cell engineering applications.

Addressing the dynamic nature of native ECM is a major challenge. ELRs, due to their ability to respond to stimuli coupled with the ability to direct cell migration, hold promise for advancing spatiotemporal guidance of cells within the matrix in more sophisticated *in vitro* models. The rational design of ELRs, incorporating selective bioactive tails into their backbone, enables the creation of cell-instructive matrices that guide cell migration and differentiation, even directing complex processes like angiogenesis. This is crucial, as creating larger and more complex *in vitro* models requires effective and functional vascularization, achievable only through matrices that effectively guide these processes.

ELRs have been applied in the fabrication of 3D models as solid scaffolds for spheroid formation, and organoids, supporting the growth and differentiation of stem cells, and acting as a substitute for Matrigel.

Additionally, ELRs exhibit excellent processability, so advanced biofabrication techniques, such as 3D bioprinting or organ-on-a-chip manufacturing, can be employed for fabricating accurate *in vitro* models. As the limited repertoire of suitable bioinks compared to the various biomaterials used in traditional tissue engineering scaffolds remains a challenge, the emergence of ELRs opens new horizons for creating models with intricate topographies that mimic native tissues using advanced techniques as 3D bioprinting and organ-on-a-chip, which remains unexplored.

In conclusion, the integration of elastin into the toolkit for *in vitro* model fabrication represents a transformative step forward in improving accuracy and efficiency. This progress extends beyond 3D bioprinting, influencing organoids and organ-on-a-chip systems, and opens exciting prospects for advancing research, drug development, and personalized medicine.
